# Breast density in NF1 women: a retrospective study

**DOI:** 10.1007/s10689-023-00355-y

**Published:** 2024-01-25

**Authors:** R. De Santis, G. Cagnoli, B. Rinaldi, D. Consonni, Beatrice Conti, M. Eoli, A. Liguori, M. Cosentino, G. Carrafiello, O. Garrone, M. Giroda, C. Cesaretti, M.S. Sfondrini, D. Gambini, F. Natacci

**Affiliations:** 1 Radiology Unit, Fondazione IRCCS Ca’ Granda Ospedale Maggiore Policlinico, Milan, Italy; 2 Medical Genetics Unit, Fondazione IRCCS Ca’ Granda Ospedale Maggiore Policlinico, Milan, Italy; 3 Epidemiology Unit, Fondazione IRCCS Ca’ Granda Ospedale Maggiore Policlinico, Milan, Italy; 4 Neurooncology Unit, Fondazione IRCCS Istituto Neurologico Carlo Besta, Milan, Italy; 5 Oncology Unit, Fondazione IRCCS Ca’ Granda Ospedale Maggiore Policlinico, Milan, Italy; 6 Breast Surgery Unit Fondazione IRCCS Ca’ Granda Ospedale Maggiore Policlinico, Milan, Italy

**Keywords:** Neurofibromatosis type 1, Breast cancer, Breast density, Screening

## Abstract

Neurofibromatosis type 1 (NF1) is an autosomal dominant condition caused by neurofibromin haploinsufficiency due to pathogenic variants in the *NF1* gene. Tumor predisposition has long been associated with NF1, and an increased breast cancer (BC) incidence and reduced survival have been reported in recent years for women with NF1. As breast density is another known independent risk factor for BC, this study aims to evaluate the variability of breast density in patients with NF1 compared to the general population. Mammograms from 98 NF1 women affected by NF1, and enrolled onto our monocentric BC screening program, were compared with those from 300 healthy subjects to verify differences in breast density. Mammograms were independently reviewed and scored by a radiologist and using a Computer-Aided Detection (CAD) software. The comparison of breast density between NF1 patients and controls was performed through Chi-squared test and with multivariable ordinal logistic models adjusted for age, body mass index (BMI), number of pregnancies, and menopausal status.breast density was influenced by BMI and menopausal status in both NF1 patients and healthy subjects. No difference in breast density was observed between NF1 patients and the healthy female population, even after considering the potential confounding factors.Although NF1 and a highly fibroglandular breast are known risk factors of BC, in this study, NF1 patients were shown to have comparable breast density to healthy subjects. The presence of pathogenic variants in the *NF1* gene does not influence the breast density value.

## Introduction

Pathogenic variants in *NF1* cause Neurofibromatosis type 1 (NF1) or Von Recklinghausen disease, an autosomal dominant disorder with an incidence between of 1:2000-1:2800 births [1, 2]. *NF1* is located on chromosome 17q11.2 and was characterized in 1990. Neurofibromin is a large multifunctional protein with a tumour-suppressor function that regulates the RAS pathway; it inhibits the Ras-GTP proto-oncogene system by converting it to Ras-GDP that subsequently becomes able to inhibit “downstream” complex protein systems (RAF/MEK/ERK and PI3K/AKT/mTOR), causing a negative modulation of cell growth. In the last few years, it has been established that neurofibromin has functions beyond the regulation of the RAS pathway alone, with tissue-specific and RAS-independent regulatory properties [3, 4].

NF1 shows a complete penetrance with wide inter-individual clinical variability (intra- and extra-familial). Approximately 50% of cases are familial, and the *NF1* pathogenic variant is inherited from one of the parents, while the remaining 50% are sporadic and occur due to *de novo* defects in *NF1* [5]. The association between NF1 and tumour predisposition has been known for a long time, in particular specific associations, such as Optic Pathway Gliomas, Malignant Peripheral Nerve Sheath Tumors, pheochromocytomas, and neuroendocrine tumours, are reported; however, only in recent years, scientific literature has documented an increased incidence of BC and reduced survival for women with NF1 [6–8]. In particular, while breast cancer risk does not differ significantly compared to women in the general population after the age 50 [9–11], Suarez-Kelly and colleagues identified a 5-fold increased risk of developing breast cancer before the age of 50 for women with NF1 [12].

Although *NF1* is considered a “driver gene” in the pathogenesis of BC also in the general population, and *NF1* haploinsufficiency is a known predictive factor of poor clinical outcome both in sporadic, and NF1-related BC, the exact mechanisms underlying the increased risk of developing BC in women with NF1 are still not clarified [13].

In 2017 National Comprehensive Cancer Network (NCCN) guidelines recommended annual breast screening for NF1 women with mammography, considering tomosynthesis, and ultrasound starting from age 30, optional breast MRI with contrast (CE-MRI) from age 30–50 [14]. More recently, the new ERN GENTURIS tumour surveillance guidelines for individuals with neurofibromatosis type 1 have recently outlined the breast tumour surveillance guideline for NF1 women, consisting of annual CE-MRI or mammography from 30 to 50 years of age [15].

Despite the overall high performance of mammography screening, its sensitivity drops significantly when screening women with dense breast [16]. Since breast density has a central role and a substantial impact on BC risk [16, 17], it should be taken into account when choosing the appropriate screening protocol.

Breast density generally varies from woman to woman due to differences in tissue composition and has a central role and substantial impact on BC risk. It also reflects the amount of breast fibroglandular tissue, composed of epithelial structures surrounded by fibroblast and connective tissue, including extracellular matrix proteins, which drives the tissue stromal architecture [18]. Notably, breast density has a much higher heritability than breast cancer itself since genetics seems to be accountable for 53 to 70% of breast density variability [19, 20]. To date, the esteem of BC incidence in dense breasts is 2–6 times higher than in lower density breasts groups, likely reflecting a higher amount of glandular tissue and a consequently reduced screening sensitivity of mammograms [21]. Interestingly, *NF1* haploinsufficiency is also known to perturb the extracellular matrix (ECM), largely contributing to breast density [22]. Our study aimed to describe breast density distribution in NF1 women and compare it with the general population. Moreover, as the choice of the most effective BC screening approach is also tailored to breast density, defining breast density in NF1 will help to improve BC surveillance in these women [22].

## Materials and methods

This is a single-center, observational case-control study. The case population comprises 98 NF1 women (with confirmed molecular diagnosis) who are part of a more extensive series of affected subjects in follow-up at our NF1 clinic. These subjects are heterogeneous in terms of age (from 18 to 73 years) and geographical origin. Data were collected from February 2018 to October 2021.

For each case, we selected up to three controls from the general population: the control cohort included 300 women (with no familial history of NF1) that performed routine screening mammography to prevent BC from June 2020 to October 2021. In both cohorts, women with a history or diagnosis of BC during the screening program have been excluded.

As part of the current district guidelines dedicated to NF1 patients (Diagnostic, Therapeutic and Care Pathways (PDTA) (web reference [23]) NF1 women between the ages of 30 and 40 are offered a bilateral breast ultrasound. At the same time, mammography is introduced from the age of 40 onwards. In specific cases (familiarity, peculiar characteristics of the breast tissue, or suspected lesions), we propose the screening program to younger women or indicate further clinical and/or instrumental examinations - e.g. Contrast-Enhanced Magnetic Resonance Imaging (CE-MRI)-, medical or surgical oncological breast evaluation).

For both cohorts, mammograms were acquired using the standard protocol adopted in our Centre, which consists in 2D cranio-caudal projection and medium-lateral-oblique projection with three-dimensional digital breast tomosynthesis and subsequent synthetic reconstruction. All eligible women completed enrolment questionnaires to collect personal information (i.e. weight and height, history of benign diseases, breastfeeding, menopausal status, and parity) [24].

Breast density was classified in four categories (A: almost entirely fatty breast, B: scattered areas of fibroglandular density, C: heterogeneously dense breast, D: extremely dense breast) according to ACR BI-RADS atlas 5th edition [25].

In order to minimize the inter-operator variability [26] breast density was first determined by a radiologist with ten years of experience in breast imaging, then compared with the examination results of digital breast tomosynthesis using CAD software of Synapse 5, FUJIFILM Medical Systems USA Inc. In case of mismatch, a second radiologist with 15 years of experience in breast imaging evaluated the exam.

The study follows the principles of 2013 revision of the Declaration of Helsinki. It was approved by the Fondazione IRCCS Ca’ Granda Ospedale Maggiore Policlinico Ethical Committee and Scientific Board (N°107–2022). All participants provided written informed consent.

### Statistical analysis

We report continuous data as means and Standard Deviations (SD), and categorical data as counts and percentages. We used Mann-Whitney and Chi-squared tests to compare quantitative and categorical data between NF1 patients and healthy controls. Since breast density is (or may be) associated with age, body mass index (BMI), reproductive history, and menopausal status, we also compared breast density between NF1 patients and controls by fitting multivariable ordinal logistic models adjusted for age (categorical), BMI, number of pregnancies, and menopausal status. Statistical analysis was performed with Stata 17 (StataCorp. 2021) [27].

## Results

The case series includes a total of 98 NF1 women and a control group of 300 healthy women. All the results are listed in Tables 1 and 2.

**Table 1:** Considered variables in the population with Neurofibromatosis 1 (NF1) and in the control cohort (Healthy)

Variable	NF1		Healthy controls		P-value
	N	%	N	%	
Total	**98**	100	**300**	100	< 0.001
Age (years), mean (DS)	**52.1 (7.9)**		**58.0 (10.6)**		< 0.001
Age					< 0.001
<50	**43**	43.9	**74 **	24.7	
50–59	**34**	34.7	**105**	35.0	
60–69	**19**	19.4	**73**	24.3	
70+	**2**	2.0	**48**	16.0	
BMI (kg/m2), mean (DS)	23.9 (4.4)		**23.8 (4.1)**		0.96
BMI					
<18.5	**6**	6.1	**16**	5.3	0.48
18.5–24.9	**61**	62.2	**174**	58.0	
25.0-29.9	**19**	19.4	**81**	27.0	
>30.0-	**12**	12.2	**29**	9.7	
N. of pregnancies					
0	**43**	43.9	**78**	26.0	0.01
1	**22**	22.5	**90**	30,0	
2	**28**	28.6	**97**	32.3	
3+	**5**	5.1	**31**	10.3	
No data	**0**	0.0	**4**	1.3	
Menopausal status					
No	**49**	50.0	**90**	30.0	< 0.001
Yes	**49**	50.0	**210**	70.0	

**Table 2:** Association between ACR and selected variables in patients with Neurofibromatosis 1 (NF1) and in the healthy control group

Variable											
	A		B		C		D		P-value*		P-value**
	N	%	N	%	N	%	N	%			
Total	**60**	15.1	**173**	43.5	**127**	31.9	**38**	9.5			
Age									< 0.001	0.23	
<50	**10**	8.5	**35**	29.9	**46**	39.3	**26**	22.2			
50–59	**19**	13.7	**63**	45.3	**49**	35.3	**8**	5.8			
60–69	**22**	23.9	**46**	50.0	**20**	21.7	**4**	4.3			
70+	**9**	18.0	**29**	58.0	**12**	24.0	**0**	0.0			
BMI									< 0.001	<0.001	
<18.5	**1**	4.5	**7**	31.8	**10**	45.5	**4**	18.2			
18.5–24.9	**22**	9.4	**100**	42.6	**80**	34.0	**33**	14.0			
25.0-29.9	**25**	25.0	**45**	45.0	**29**	29.0	**1**	1.0			
30.0+	**12**	29.3	**21**	51.2	**8**	19.5	**0**	0.0			
N. pregnancies									0.43	0.92	
0	**19**	15.7	**43**	35.5	**45**	37.2	**14**	11.6			
1	**18**	16.1	**52**	46.4	**28**	25.0	**14**	12.5			
2	**17**	13.6	**56**	44.8	**43**	34.4	**9**	7.2			
3+	**6**	16.7	**19**	52.8	**10**	27.8	**1**	2.8			
No data	**0**	0.0	**3**	75.0	**1**	25.0	**0**	0.0			
Menopausal st.									< 0.001	0.01	
No	**12**	8.6	**42**	30.2	**57**	41.0	**28**	20.1			
Yes	**48**	18.5	**131**	50.6	**70**	27.0	**10**	3.9			
NF1									0.27	0.72	
Controls	**50**	16.7	**130**	43.3	**95**	31.7	**25**	8.3			
NF1	**10**	10.2	**43**	43.9	**32**	32.7	**13**	13.3			
NF1 (< 50years)									0.89	0.72	
Controls	**6**	8.1	**24**	32.4	**28**	37.8	**16**	21.6			
NF1	**4**	9.3	**11**	25.8	**18**	41.9	**10**	23.3			

*From chi-squared tests

**From a multivariate ordinal logistic model containing all the covariates

NF1 patients were on average 6 years younger than healthy controls (Table 1). In particular, 44% of patients with NF1 were below 50 years compared to 25% of healthy subjects. BMI was almost identical in the two groups, while NF1 patients had a lower number of pregnancies, and a low frequency were in menopausal status.

In univariate analyses we observed clear inverse associations between ACR, age, BMI, and menopausal status (Table 2). Associations with age and BMI were confirmed in a multivariable analysis. NF1 was no associated with ACR, either overall or when we restricted analysis to women aged < 50 years at mammography.

## Discussion

NF1 is a complex neuroectodermal disease characterized by autosomal dominant inheritance, high penetrance, wide variability in expression, and a multisystemic involvement; neoplasms are the most common cause of death in such patients and cause a reduction in life expectancy that can be up to 10–15 years shorter than in the general population [28].

The correlation between NF1 and the risk of developing breast cancer has become increasingly evident in recent years. From the first case report referring to such a possible association, in 1933, an increasing number of works have been published, allowing to determine a real correlation between NF1 and the risk of developing BC [8]. Recent studies have estimated that NF1 women have an up to five-times increased risk of developing this complication before the age of 50 and, in general, a risk of developing breast cancer (especially invasive ductal-type carcinomas) increased at least 3-fold compared to the general population [12].

In women with NF1, breast density has not been addressed yet, however this information is of particular importance, given the correlation between the degree of breast density and the risk of developing malignant breast cancer, recognised in the general population [17, 29, 30].

So far, only a few studies have investigated breast density correlation with high penetrant BC conditions, with heterogeneous results. For example, in their work of 2010, Passaperuma and colleagues [31] did not find any correlation between breast density grade and breast cancer risk in women carrying BRCA1/2 pathogenetic variants, while recently Han and colleagues [32] found that higher breast density is associated with having a positive breast cancer familial history in premenopausal women, data also reported by Ziv et al. [33].

Our study is the first to our knowledge that focuses on breast density in NF1 women compared to that of the general population; we found no significant differences in breast density between the two cohorts, even after adjusting the data for potential confounders (age, BMI, number of pregnancies, and menopausal status). Therefore, according to our results, breast density seems not to be affected by the presence of pathogenetic variants in NF1. This result is in accordance with genome-wide association studies - aimed at identifying loci associated with breast density- disclosed more than 30 significant loci, among which NF1 is not comprised [34].

Since a limiting factor in the radiological diagnosis of BC in NF1 patients is the possible presence of benign lesions, such as breast neurofibromas, that may lead to potential diagnostic doubts in the standard radiological diagnostic process [35, 36] radiologist should be trained in the study of NF1 woman mammary gland, in order to minimize the potential misinterpretations given by the possible presence of cutaneous neurofibromas at the breast level.

In conclusion our findings demonstrate that the characteristics of the breast gland of patients with NF1 are suitable to be also studied with mammography. Mammography can be considered a valid and reliable tool to prevent death for BC in women with NF1. It can be a possible alternative to MRI, constantly considering all the characteristics and variables that can influence the risk, such as familiarity or age, and the technique to be considered the most appropriate.

## Conclusion

The results of this study demonstrate that women with NF1 have a breast density comparable to that of the general population, allowing us to hypothesise that the increased BC risk in NF1 does not rely on a higher breast density. These results confirm the adequateness of the screening program proposed in the recent GENTURIS Guidelines, aimed at guarantee early breast cancer diagnosis in this high-risk population.

## Abbreviations

BC: Breast cancer
NF1: Neurofibromatosis type 1
*NF1*: Neurofibromin gene
SD: Standard Deviations
BMI: Body Mass Index
CE-MRI: Contrast-Enhanced Magnetic Resonance Imaging
